# Herpesvirus Nuclear Egress across the Outer Nuclear Membrane

**DOI:** 10.3390/v13122356

**Published:** 2021-11-24

**Authors:** Richard J. Roller, David C. Johnson

**Affiliations:** 1 Microbiology & Immunology, University of Iowa, Iowa City, IA 52242, USA; richard-roller@uiowa.edu; 2 Molecular Microbiology & Immunology, Oregon Health & Science University, Portland, OR 97239, USA

**Keywords:** de-envelopment, phosphorylation, nuclear envelopment complex, membrane fusion, hemi-fusion

## Abstract

Herpesvirus capsids are assembled in the nucleus and undergo a two-step process to cross the nuclear envelope. Capsids bud into the inner nuclear membrane (INM) aided by the nuclear egress complex (NEC) proteins UL31/34. At that stage of egress, enveloped virions are found for a short time in the perinuclear space. In the second step of nuclear egress, perinuclear enveloped virions (PEVs) fuse with the outer nuclear membrane (ONM) delivering capsids into the cytoplasm. Once in the cytoplasm, capsids undergo re-envelopment in the Golgi/trans-Golgi apparatus producing mature virions. This second step of nuclear egress is known as de-envelopment and is the focus of this review. Compared with herpesvirus envelopment at the INM, much less is known about de-envelopment. We propose a model in which de-envelopment involves two phases: (i) fusion of the PEV membrane with the ONM and (ii) expansion of the fusion pore leading to release of the viral capsid into the cytoplasm. The first phase of de-envelopment, membrane fusion, involves four herpes simplex virus (HSV) proteins: gB, gH/gL, gK and UL20. gB is the viral fusion protein and appears to act to perturb membranes and promote fusion. gH/gL may also have similar properties and appears to be able to act in de-envelopment without gB. gK and UL20 negatively regulate these fusion proteins. In the second phase of de-envelopment (pore expansion and capsid release), an alpha-herpesvirus protein kinase, US3, acts to phosphorylate NEC proteins, which normally produce membrane curvature during envelopment. Phosphorylation of NEC proteins reverses tight membrane curvature, causing expansion of the membrane fusion pore and promoting release of capsids into the cytoplasm.

## 1. Introduction

**Figure 1:**
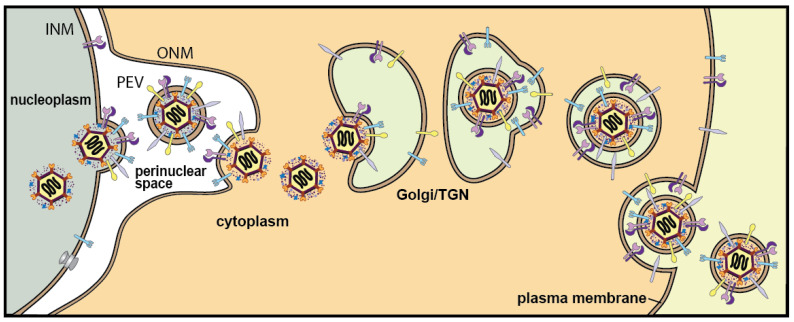
Envelopment/de-envelopment model of herpesvirus egress from cells. Capsids in the nucleus acquire an envelope from the INM by budding into the perinuclear space. The PEV envelope fuses with the ONM delivering capsids into the cytoplasm. These capsids bud into cytoplasmic membranes. Vesicles containing mature virions fuse with the plasma membrane delivering virions to the extracellular space.

Herpesviruses construct their nucleocapsids and fill them with DNA in the nucleus. These large virus particles then face the difficult challenge of crossing the nuclear envelope (NE), a structure that is not designed to allow large objects to pass. Decades ago, there was controversy, with one camp suggesting that herpes simplex virus (HSV) acquires an envelope by budding into the inner nuclear membrane (INM) and that these enveloped particles then acquire a second membrane at the outer nuclear membrane (ONM) [1]. These vesicles were thought to then ferry the enveloped virions through the cytoplasm to the plasma membrane. Others suggested that herpesvirus capsids can squeeze through impaired nuclear pores despite the large size of these particles [2]. A third model, originally proposed by Stackpole [3], suggested that herpesviruses cross the NE via a two-step process involving capsid envelopment at the INM followed by de-envelopment at the ONM (see Figure 1). The vast majority of the evidence now supports this third model, often known as envelopment/de-envelopment (reviewed in [4]). In the first step of this pathway, viral proteins disrupt the nuclear lamina then nucleocapsids interact with the INM that is wrapped around the capsids as enveloped particles bud into the space between the INM and ONM, also known as the perinuclear space. This envelopment step involves the well-characterized nuclear envelopment complex (NEC) proteins HSV UL31 and UL34, which have been extensively reviewed [4,5,6,7]. Enveloped particles found in the perinuclear space are known as perinuclear virions (PEVs). In the second step of nuclear egress, the envelopes of PEVs fuse with the ONM so that capsids with tegument proteins bound to their surfaces disengage from the ONM and move into the cytoplasm. Later these capsids acquire another membrane by budding into the Golgi or trans-Golgi network followed by delivery to the plasma membrane.

Capsid de-envelopment at the ONM consists of at least two phases. The first phase involves fusion of the PEV membrane with the ONM. This process may involve an initial phase in which the lipids in outer leaflet of the two membranes mix to produce hemi-fusion, followed by mixing of the lipids in the inner leaflets of the membranes to produce full membrane fusion and a fusion pore between PEV and the cytoplasm. The second, easily overlooked phase of this process involves expansion of the fusion pore and release of the capsid into the cytoplasm. Small fusion pores between membranes can theoretically resolve either by expansion or closure of the pore [8]. With herpesviruses, we have the example of the NEC proteins that have the capacity to alter the curvature of membranes in order to promote envelopment, the opposite of de-envelopment [4,6]. Reversing this process of curving membranes, which involves removing or altering the NEC present in PEV, might lead to expansion of the fusion pore and capsid release into the cytoplasm.

While there is extensive mechanistic information about the first step of nuclear egress, envelopment, much less is known about the viral machinery that promotes the second step, de-envelopment. In this review, we describe what is known about herpesvirus egress across the ONM, with much of the information from studies of herpes simplex virus (HSV). Given that this is such a fundamentally important and basic process, we assume that other herpesvirus families may also utilize similar mechanisms for this egress. However, we hasten to acknowledge that molecular mechanisms involved in de-envelopment remain sketchy, and thus there is ample conjecture, as well as some theoretical models attempting to explain puzzles represented by certain observations. Most of what is known about de-envelopment comes from studies of HSV and the related alpha-herpesvirus pseudorabies virus (PRV), and our review focuses on these two viruses. It would seem that such a basic process of virus egress should involve similar proteins in beta- and gamma-herpesviruses, although there are major differences between HSV and PRV. Moreover, little to nothing is known about de-envelopment with beta- and gamma-herpesviruses. The review is broken down into two sections. The first focuses on four membrane proteins: gB, gH/gL, gK and UL20, which apparently participate in the membrane fusion phase of de-envelopment. The second section focuses on the second phase of de-envelopment involving the US3 protein, a threonine/serine kinase that phosphorylates NEC proteins so as to potentially reverse effects of NEC proteins and promote fusion pore expansion and capsid entry into the cytoplasm.

## 2. HSV Membrane Proteins That Promote De-Envelopment

### 2.1. HSV Glycoproteins gB and gH/gL in Fusion of PEVs with the ONM

**Figure 2:**
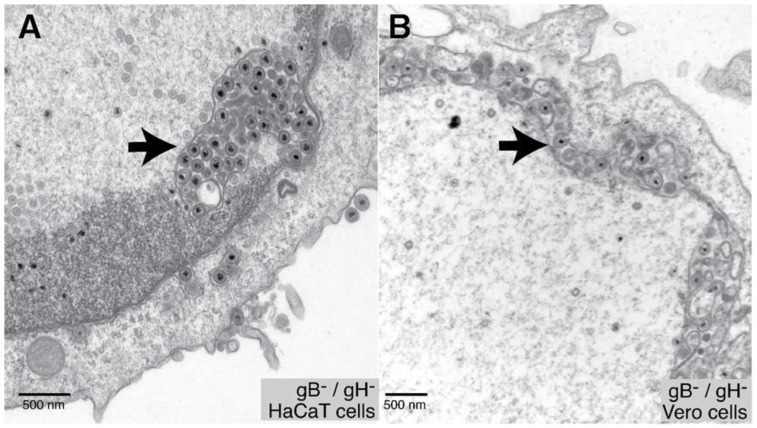
Herniations and PEVs. (**A**) HaCaT keratinocytes infected with an HSV gB^−^/gH^−^ double mutant display herniations (arrow). These vesicle membranes are continuous with the INM. (**B**) Vero cells infected with the HSV gB^−^/gH^−^ mutant display perinuclear enveloped particles (PEVs) (arrow).

A role for herpes simplex virus (HSV) glycoproteins in de-envelopment at the ONM was suggested by the observations that a mutant lacking two glycoproteins, gB and gH, exhibited defects in this step [9]. Enveloped virions accumulated in the perinuclear space or in membrane vesicles containing enveloped virions known as herniations (Figure 2). Loss of both gB and gH in HaCaT human keratinocytes produced large herniations, extensions of the INM extending into the nucleoplasm containing enveloped virions (Figure 2A) [9]. Most of the total enveloped particles in these cells were present as PEVs or in herniations, and there were substantial reductions in cell surface and cytoplasmic particles. In monkey Vero cells, there were fewer herniations and instead more extensive accumulation of enveloped virions in the perinuclear space which was often ballooned (Figure 2B).

Both gH and gB are components of the mature virus particle and are essential for the membrane fusion events that initiate virus infection (reviewed in [10,11]). It should be noted that the gH glycoprotein is found in a complex with a second smaller glycoprotein gL that is not membrane anchored [12,13]. Without gL, HSV gH is not folded normally, does not leave the endoplasmic reticulum, and is not incorporated into the mature virion envelope. Observations with the gB^−^/gH^−^ double mutant suggested that gB and gH/gL act in a manner that bears similarities to their role in virus entry, where these proteins mediate fusion of the virion envelope with host membranes. We describe data in the section below suggesting that both gB and gH/gL can affect lipid mixing and, perhaps, promote membrane fusion. Thus, in the absence of both gH/gL and gB, fusion between the virion envelope and the ONM is blocked, and enveloped particles accumulate in the perinuclear space or back up into the nucleoplasm in herniations. Consistent with this model, both gB and gH/gL were detected in nuclear membranes and in PEVs by immunofluorescence and immunoEM [9]. Others have also shown intense staining of the NE and ER by gB-specific antibodies [14].

Mutations in the gB fusion loops abolished the capacity of gB to function in de-envelopment when expressed in an HSV recombinant unable to express gH [15]. gB fusion loops are thought to play a direct role in membrane fusion by inserting hydrophobic peptide sequences into cellular membranes [16]. HSV recombinants expressing gB with any one of four fusion loop mutations (W174R, W174Y, Y179K and A261D) were unable to enter cells. In addition, all four fusion loop mutants expressed in viruses lacking gH accumulated PEVs, and there were fewer enveloped virions on cell surfaces [15]. These results support the hypothesis that gB functions directly in membrane fusion to promote nuclear egress, rather than in other processes such as binding receptors or other viral proteins. The observations also decreased the likelihood that the original HSV gB^−^/gH^−^ null mutant possessed gross defects in the virion envelope making it unable to cross the NE.

The herniations observed with the HSV gB^−^/gH^−^ mutant appeared similar to those previously observed in cells infected with HSV US3-null mutants [17,18]. HSV US3 is a viral serine/threonine kinase and is reviewed in the second section of this paper. US3 directly phosphorylated the gB cytoplasmic (CT) domain in in vitro assays, as well as in extracts of cells [19,20]. Deletion of gB in the context of a US3-null virus did not add substantially to defects in nuclear egress. The majority of the US3-dependent phosphorylation involved amino acid T887 in the cytoplasmic domain of gB, which is present in a motif similar to that recognized by US3 in other proteins. HSV recombinants expressing a gB substitution T887A or a gB truncated at residue 886 and lacking gH accumulated in the perinuclear space and in herniations [19]. These observations supported the conclusion that phosphorylation of the gB CT domain is important for gB-mediated fusion with the ONM. US3 is incorporated into the tegument layer (between the capsid and envelope) of HSV virions, and this close proximity to the gB CT tail might lead to phosphorylation and triggering of gB^-^ mediated fusion. Alternatively, US3 phosphorylation of gB might control the incorporation of gB into the PEV membrane. Related to this possibility, PEV particles that accumulate with an HSV US3-null mutant were characterized by cryo-electron microscopy and found to contain few glycoprotein spikes [21].

### 2.2. Caveats and Puzzles Associated with Observations That gB and gH/gL Promote De-Envelopment

The observations that gB and gH/gL participate in de-envelopment bear similarities to the process of virus entry into cells. However, there is a big difference. Models for entry of HSV and essentially all herpesviruses suggest that various forms of gH/gL interact with gB to cause gB-mediated cell fusion (reviewed in [10,22,23]. However, evidence for direct interactions between these two glycoproteins is rather thin for most herpesviruses (see discussion in [24]). HSV virus entry begins with glycoprotein gD binding to cellular receptors, then gD apparently interacts with and alters gH/gL [25]. Following this, gH/gL is said to interact with gB, which is clearly a membrane fusion protein based on its structure. Pre- and post-fusion structures of gB are similar to the pre- and post-fusion forms of the vesicular stomatitis virus G protein, a type III fusion protein that promotes VSV entry [26,27,28]. HSV mutants lacking any one of gD, gH/gL or gB are unable to enter cells [13,29,30,31]. In contrast, it was necessary to mutate both gB and gH to substantially reduce nuclear egress. However, it should be noted that the numbers of PEVs observed with a gB^-^ mutant (expressing gH/gL) were about triple those observed with w.t. HSV in HaCaT cells, though much more major defects (30X more PEVs) were observed with gB^−^/gH^−^ double mutant [9]. These results suggest that gH/gL, without gB, can mediate some level of de-envelopment.

**Figure 3:**
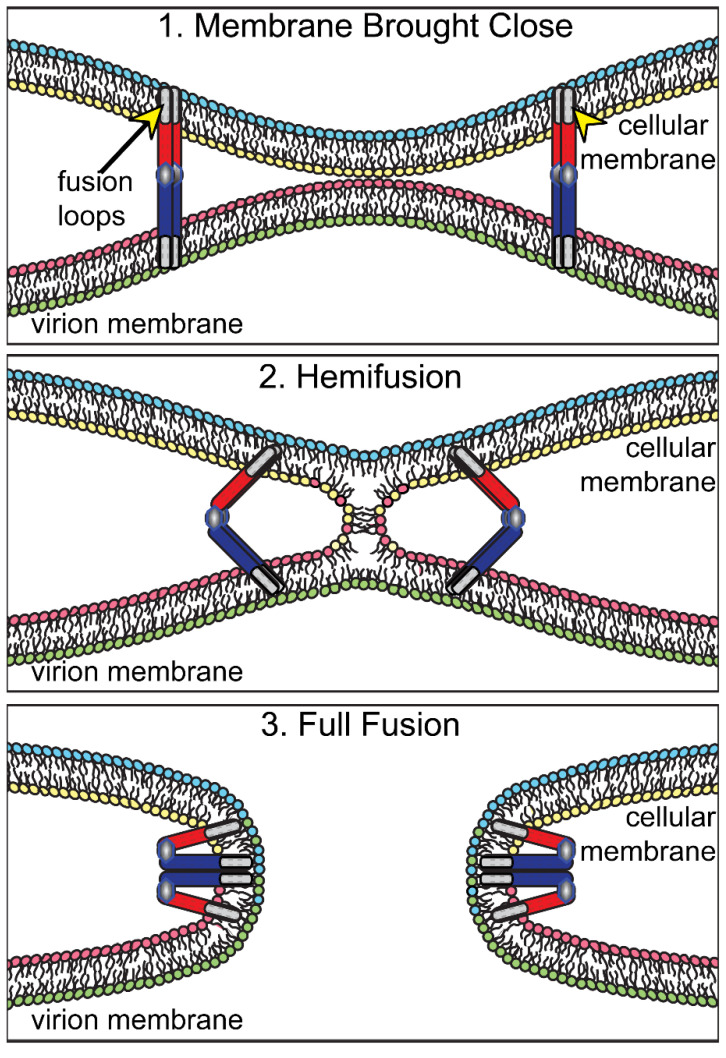
Model of virion envelope fusion with cellular membranes. (**1**) The virion envelope is brought close to a cellular membrane by the action of viral fusion proteins (illustrated to represent any viral fusion protein). Fusion loops in the fusion protein insert into the cellular membrane. (**2**) The viral fusion protein, acting like a hinge, pulls the two membranes close together, promoting hemi-fusion, i.e., mixing of the lipids in the outer leaflets. (**3**) Continued folding back of the fusion proteins produces mixing of lipids in the inner leaflets of the two membranes creating a fusion pore.

To explain these differences, there is some evidence that gH/gL might interact directly with membranes. There were reports that an alpha-helical peptide domain of gH (in the region of a.a. 626–644) can interact with membranes and peptides can inhibit cell–cell fusion and virus entry [32,33,34]. Other studies involving the entire gH/gL protein supported the hypothesis that gH/gL can induce membrane hemi-fusion, which was first described as the first step in influenza virus HA-mediated entry fusion [35]. Hemi-fusion involves mixing of the outer leaflets of two different membranes, but not the mixing of the inner leaflets of these membranes (see Figure 3). Subramanian et al. used assays involving the transfer of the lipid ganglioside GM1, which was present in the outer leaflet of the plasma membrane of Vero cells, into the surface membranes of CHO cells, which do not express GM1 [36]. Expression of gD (to bring membranes close) and gH/gL in Vero cells produced hemi-fusion. However, complete fusion of cells, detected by transfer of soluble GFP from the cytoplasm of Vero cells into CHO cells, was produced by expression of gD, gH/gL and gB. These data produce strong support for the conclusion that gH/gL can interact directly with membranes, perturbing the lipids sufficiently so that the outer leaflets of two opposing membranes mix with one another. This membrane mixing activity of gH/gL may be sufficient to promote de-envelopment at the ONM.

It is tempting to think of de-envelopment fusion as analogous to virus entry and therefore likely to be mediated by fusion proteins embedded in the PEV envelope that are activated in response to interactions with some component of the ONM. This seems most likely given how gB and gH/gL function in virus entry, and these proteins might also promote de-envelopment in this manner. However, it is also possible that the fusion machinery (gB and gH/gL or other proteins) might reside in the ONM and be activated by interaction with some component of the PEV envelope. Current evidence does not distinguish between these two possibilities. Moreover, there is no information on which cellular or viral proteins might trigger the viral fusion machinery.

A second puzzle relates to observations that another herpesvirus does not appear to rely on gB and gH/gL for de-envelopment. All herpesviruses express gB and gH/gL proteins, and thus it might seem likely that all might use these glycoproteins in this fundamental process. Nevertheless, porcine pseudorabies virus (PRV) mutants lacking gB and gH, gB and gD, gD and gH or gH and gL all showed no defects in virus egress [37]. In addition, immunoEM studies involving both monoclonal and polyclonal antibodies failed to detect PRV glycoproteins in the INM or in PEV. These PRV immunoEM studies differ from the immunoEM results from several laboratories that demonstrated HSV glycoproteins gB, gD, gH/gL and gM present in the INM and perinuclear virions [9,38,39], and gD was observed in PEVs purified from infected cells and subjected to Western blotting [40]. Moreover, HSV gB and gH/gL display intense immunofluorescence in nuclear and ER membranes, more intense than that observed in the plasma membrane [9,14]. These differences between HSV and PRV must be considered in light of other major differences in egress pathways of egress for these viruses. For example, PRV mutant lacking gM and gE/gI showed major defects in secondary envelopment, i.e., unenveloped virions accumulated in the cytoplasm [41]. In contrast, an HSV mutant lacking both gM and gE was only marginally compromised in virus replication [42]. Moreover, an HSV mutant lacking both gB and gD was found to be defective in secondary envelopment [43], but that was not the case with a PRV gB^−^/gD^−^ double mutant [37]. There are also major differences in how PRV and HSV egress into neuronal axons (reviewed in [44]).

### 2.3. gK and UL20, Other Membrane Proteins That May Regulate De-Envelopment Fusion

HSV-1 gK is a ~40 kDa glycosylated multi-pass membrane protein that is the encoded by the UL53 gene [45]. gK is essential for virus replication, and gK null mutants exhibited defects in secondary envelopment, shown by accumulation of unenveloped or partially enveloped capsids in the cytoplasm and far fewer cell surface virions [46,47]. Point mutations in gK represent, by far, the most frequent genetic mutations that produce the syncytial phenotype in which infected cells fuse extensively in early stages of infection [48,49,50,51,52,53]. This cell–cell fusion is apparently mediated by gB, the viral fusion protein [54]. It had long been hypothesized that gK might negatively regulate the capacity of gB to fuse membranes [55,56,57,58].

The involvement of gK in nuclear egress was indicated by the surprising observations that overexpression of gK in stably transfected cells followed by infection of wild-type HSV resulted in extensive accumulation of enveloped virus particles in the perinuclear space [46]. There were also very few virus particles in the cytoplasm or on cell surfaces in these cells. Consistent with an involvement in nuclear egress, gK displayed subcellular localization consistent with a function in nuclear egress. gK peptide-specific antibodies showed that gK was not on cell surfaces, but accumulated extensively in the ER and NE [56]. Moreover, gK N-linked oligosaccharides remained in the high mannose form, consistent with an inability to be transported to the Golgi apparatus and suggesting that most gK is retained in the ER and NE [56]. Subsequent studies of gK with insertions of a 14 a.a. epitope produced evidence that some gK reached cell surfaces [59]. Unfortunately, these studies did not compare to the anti-gK antibodies used in the earlier studies, and thus it is possible that the insertion of 14 a.a. into gK altered its traffic in cells. In contrast to the overexpression of gK, the loss of gK by deletion caused defects in secondary envelopment [46,47]. Given the hypothesis that HSV gK negatively regulates the capacity of gB to fuse membranes, the observations of marked accumulation of PEVs with overexpression of gK supports a model in which gK reduces gB-mediated membrane fusion between the virus and the ONM. However, it is not clear whether overexpression of gK also impairs gH/gL in the de-envelopment process.

HSV UL20 may be another player in the de-envelopment process. UL20 is a ~24 kDa multi-pass transmembrane protein, but, unlike gK, UL20 is not glycosylated [60,61]. UL20 also extensively accumulated in the NE and ER, with little of the protein present on cell surface membranes [61]. UL20 interacted with gK, and the two proteins affect the traffic of one another in transfected cells [62,63]. Early studies involving an HSV UL20 deletion mutant described marked accumulation of enveloped virions in the perinuclear space [64]. However, this UL20 mutant did not just contain a simple deletion of the entire UL20 open reading frame, but instead the virus expressed a C-terminal fragment of the UL20 protein fused in frame to the UL20.5 protein [65]. The UL20.5 protein also accumulates within nuclear membranes [66]. Subsequently, an HSV deletion mutant lacking all UL20 sequences and without effects on the UL20.5 gene did not exhibit these defects in nuclear egress [65]. Instead, this UL20 mutant showed defects in secondary envelopment, so that cytoplasmic capsids accumulated in the cytoplasm, similar to the defects seen with the HSV gK- null mutant [46]. A PRV UL20 mutant also showed defects in cytoplasmic assembly, accumulating vesicles containing numerous enveloped virions [67]. Therefore, the retention of HSV enveloped virions in the perinuclear space observed with the first HSV UL20 mutant [64] was apparently due to expression of the UL20–UL20.5 fusion protein, not loss of UL20. Nevertheless, the phenotype of this UL20/UL20.5 fusion protein appears similar to that of gK overexpression, causing reduced de-envelopment.

There are also important relationships between gK and UL20 and gB. Syn mutations most frequently affect the UL53 (gK) gene, but mutations altering the gB cytoplasmic domain or the UL20 protein also produce the syncytial phenotype [13,48,49,50,51,52,53]. Moreover, there was a report that gK and UL20 interact with gB [58]. In addition, coexpression of UL20 and gK blocks cell–cell fusion mediated by transfecting cells with gD, gH/gL and gB [68]. These results, when coupled with observations of gK overexpression and the UL20–UL20.5 fusion protein, support the hypothesis that gK and UL20 negatively regulate gB in de-envelopment fusion.

### 2.4. Summary of the Roles of HSV Membrane Proteins gB, gH/gL, gK and UL20 in Nuclear Egress

Mutants lacking both gB and gH accumulated enveloped particles in the perinuclear space or herniations. gB proteins with mutations in the fusion loops or in cytoplasmic residues phosphorylated by US3 when combined with a gH-null mutant also displayed these defects. To explain the observations that both gB and gH must be deleted in order to substantially reduce de-envelopment, it appears that both gB and gH/gL may have the capacity to cause lipid mixing. Related to the role of gB in de-envelopment, there are two other membrane proteins gK and UL20 that are thought to interact with gB and might negatively regulate gB in membrane fusion in cells. All three of UL20, gK and gB are mutated in HSV syn mutants. It seems more than coincidence that all three of these proteins are involved in cell–cell fusion and mutant forms of these proteins can alter de-envelopment. It also makes ample sense that herpesviruses construct fusion machinery including gB and gH/gL in order to enter cells and could also use this machinery for the similar process of fusing the virion envelope with the ONM. That said, there is also good evidence that there are other mechanisms by which HSV and other herpesviruses cross the ONM (see Section 3 below).

## 3. US3-Mediated Phosphorylation of NEC Proteins in De-Envelopment

### The Role of US3 in De-Envelopment

The first indication that alpha-herpesvirus proteins promote de-envelopment involved a PRV mutant lacking that US3 gene that accumulated PEVs [69]. These enveloped virions were not distributed throughout the perinuclear space but were found in herniations of the inner nuclear membrane [17,69,70], similar to the herniations observed later with gB^−^/gH^−^ double mutants [9] (see examples in Figure 2). US3 mutations in other, distantly related alpha-herpesviruses also produced similar defects in de-envelopment [71,72,73,74]. Therefore, pUS3 is important for efficient de-envelopment of alpha-herpesvirus capsids at the ONM.

US3 is a serine/threonine protein kinase and is conserved among the alpha-herpesviruses, but not in beta- and gamma-herpesviruses. It has been reported to phosphorylate numerous viral and cellular substrates in the infected cell and to regulate infected cell properties as diverse as protection from apoptosis, promotion of translation and inhibition of antigen presentation (reviewed in [72]). The de-envelopment function of HSV pUS3 requires its kinase activity, since point mutations that ablate kinase activity have the same de-envelopment phenotype as a US3 deletion [17]. This suggests that pUS3 functions in de-envelopment by phosphorylation of other protein substrates. US3 is a structural component of the PEVs, but whether its de-envelopment function requires incorporation into the virion is not known [75].

The critical question about US3 function in de-envelopment is the identity of the relevant protein substrate(s). The available data are consistent with two non-exclusive hypotheses. The first hypothesis involves US3 phosphorylation of gB, and perhaps gH/gL, so that this machinery, which causes fusion of the PEV and ONM membranes, is given increased fusogenic activity by virtue of phosphorylation. Evidence of this hypothesis is summarized above.

**Figure 4:**
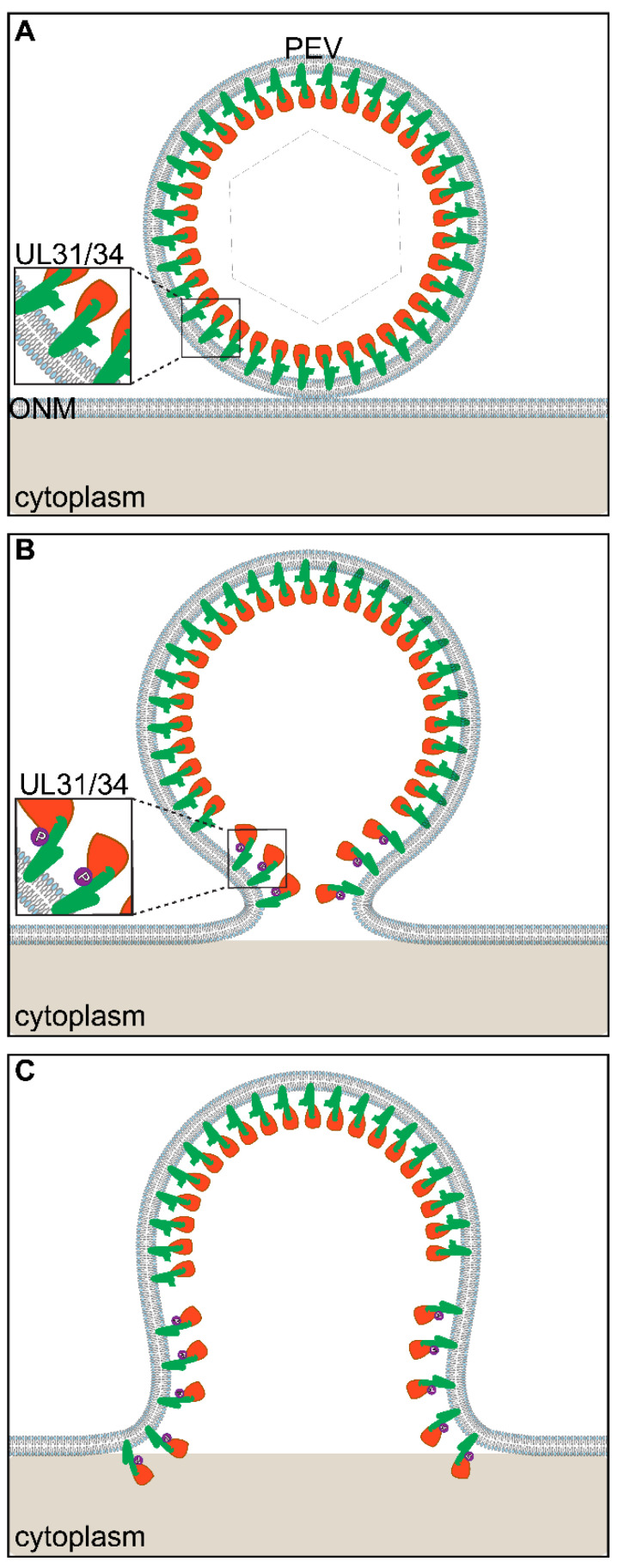
Model for fusion pore expansion by phosphorylation of the NEC. (**A**) Membrane curvature in the PEV is induced and maintained by interactions between NEC heterodimers embedded in the lipid bilayer. (**B**) Upon opening of a fusion pore, pore expansion can be driven by US3-mediated phosphorylation of UL31 N-terminal domain near the membrane proximal region of the heterodimer. (**C**) UL31/UL34 continues to be altered in conformation and moves into the cellular membrane further relaxing membrane curvature and allowing expansion of the fusion pore.

The second hypothesis is that pUS3 phosphorylation regulates the reversal of the tight membrane curvature brought about by the presence of the NEC (UL31/UL34 proteins), so that there is an expanded fusion pore and release of unenveloped capsids into the cytoplasm (see Figure 4). Evidence in favor of this second hypothesis comes from the observation that mutation of pUS3 phosphorylation sites on one of the NEC proteins produces a de-envelopment defect that is similar to that seen with US3 mutants [18]. HSV-1 US3 phosphorylates both UL34 and UL31 [17,18,76], but phosphorylation of UL34 is not conserved in PRV [70]. HSV-1 UL34 is phosphorylated in its flexible C-terminal stalk and UL31 in its N-terminal domain [17,18,76]. The function of UL34 phosphorylation in HSV-1 is unclear, since mutation of the phosphorylation site has no effect on virus replication or nuclear egress [17]. In contrast, phosphorylation of UL31 at US3-specific phosphorylation sites is required for efficient virus replication and nuclear egress. Replacement of UL31 phosphorylated residues with alanines resulted in a de-envelopment defect and diminished single-step replication similar to that seen for US3 mutant viruses [18]. Replacement of the phosphorylated residues with the phosphomimetic glutamic acid resulted in a replication defect, and a nuclear egress defect that precedes de-envelopment, i.e., capsids accumulated in the nucleus and accumulation of PEVs, was not observed. These observations suggest that UL31 is a critical substrate for the de-envelopment function of US3. UL31 might participate directly in de-envelopment in a manner regulated by pUS3 or, alternatively, phosphorylated UL31 might recruit some other de-envelopment factor. All alpha-herpesviruses encode UL31 homologs with serine or threonine residues in the context of basic residues, motifs that are similar or identical to HSV and PRV US3 phosphorylation motifs.

One mechanism by which US3-mediated phosphorylation of UL31 might promote de-envelopment involves altering the capacity of the NEC to induce and maintain membrane curvature. By releasing the UL31/34 imposed curvature of the virion envelope that is imposed at the INM, the fusion pore may expand during de-envelopment and allow release of the capsid into the cytoplasm. To understand this better it is worth reviewing how UL31 and UL34 mediate membrane curvature during envelope at the INM. UL31 and UL34 expressed in transfected cells (without other viral proteins) promote vesicle formation in the perinuclear space [77]. The crystal structures of UL31/UL34 heterodimers suggest assembly into hexameric arrays similar to those observed in vesicles formed by UL31 and UL34 in vitro [78,79,80,81,82]. Mutations in UL31 and UL34 that disrupt hexamer formation depress membrane budding in vitro and produce a budding defect in infected cells [78]. This suggests that association of UL31/UL34 heterodimers into hexameric arrays is important for membrane budding, perhaps by inducing lipid ordering that favors curvature [83]. Evidence that pUS3 regulates the association state of UL31/UL34 heterodimers comes from observations that US3 mutants form NEC aggregates in the absence of capsid budding and that these aggregates correspond to deformations of the nuclear membrane and areas of tight membrane curvature [84]. Thus, the NEC can produce tight curvature of the INM, and this is regulated by pUS3. Our model illustrated in Figure 4 would suggest that reversal of the NEC-imposed curvature at the ONM during de-envelopment is necessary to permit expansion of the fusion pore so that the capsid can be released into the cytoplasm. Without reversal of NEC-imposed curvature, the fusion pore may close, regenerating a fully enveloped PEV. US3 would function in this model by phosphorylating UL31 to alter the structure of UL31/34, thereby reversing membrane curvature.

The HSV UL21 protein also plays a significant role in de-envelopment as shown by the accumulation of PEVs in cells infected with a UL21 deletion virus [85]. Indeed, the PEV accumulations appear quite similar to those seen in US3 mutant-infected cells, and mutation of UL21 also causes formation of punctate NEC aggregates in the nuclear envelope similar to those seen with US3 mutants [85,86]. These observations suggest that UL21 and US3 are on the same pathway for promoting de-envelopment or have overlapping functions. The situation, however, is complex. UL21 functions as a phosphatase adapter, recruiting protein phosphatase 1 (PP1) by way of a sequence motif that is conserved across the alpha-herpesviruses [86]. Deletion of UL21 or mutation of its phosphatase adapter motif results in increased phosphorylation of some US3 substrates, including UL31 [86]. Furthermore, mutation of the UL21 PP1 adapter motif results in a virus replication defect that can be partially suppressed by mutations in US3 that may affect its catalytic activity [86]. These results suggest that, in some circumstances, US3 and UL21 activities are opposed to one another. How these results might be reconciled is not clear at this point, but it is possible that UL21 may function differently in the envelopment and de-envelopment steps of nuclear egress. UL21 associates with intranuclear capsids [87,88], and Benedyk et al. have suggested that capsid-associated UL21 might locally promote dephosphorylation of UL31 and thereby promote NEC assembly and virion budding into the perinuclear space [86]. US3 phosphorylation of UL31 in areas where capsids are not docked might prevent NEC self-association and inhibit capsid-less budding events. At the de-envelopment step, UL21 and US3 might cooperate in a way that may not involve the phosphatase adapter activity of UL21. However, these results again highlight the important role of phosphorylation and, perhaps, de-phosphorylation in regulating de-envelopment.

## 4. Models for HSV De-Envelopment

The observations reviewed here suggest a consensus model for HSV de-envelopment in which gB and gH/gL can each independently promote the membrane fusion phase of de-envelopment and pUS3 both regulates the de-envelopment activity of gB and promotes the fusion pore expansion/capsid release phase. The two multi-span proteins gK and UL20 may also contribute to regulating gB, and perhaps gH/gL, in this process. It is important to note, however, that neither loss of both gB and gH nor loss of US3 completely blocks HSV nuclear egress; there were still significant quantities of cell surfaces virions [17,19]. With respect to gB and gH/gL, in Vero cells, wild-type HSV produced 85% cell surface and 5% PEVs, while the gB^−^/gH^−^ mutant produced 20% cell surface and 59% PEVs. Thus, there were approximately 4–5 fold reductions in cell surface particles and 10 fold increases in PEVs. With loss of US3 there were defects in growth that are far more modest than those associated with loss of either UL31 or UL34. This suggests that nuclear egress proceeds relatively efficiently even without US3 function [17,70,89,90,91,92,93]. Indeed, the fraction of the US3 deletion growth defect that is due to its function in nuclear egress is small, since adding a US3 inactivating mutation onto an NEC deletion gives a further growth defect similar in magnitude to the US3 mutation on a wild-type background [84]. Therefore, there appear to be viral or cellular proteins, in addition to US3 and gB and gH/gL, that promote de-envelopment. These other viral or cellular proteins might also account for the de-envelopment observed with the PRV gB^−^/gH^−^ double mutant [37].

EM images of cells infected with wild-type HSV show relatively few PEVs; instead, the majority of enveloped particles accumulate on cell surfaces. Thus, the de-envelopment process is rapid. However, in US3^-^ or gB^−^/gH^−^ mutant virus-infected cells, there is substantial accumulation of PEVs over time, even though some virus reaches the cytoplasm and cell surfaces. This delay continues until long after infection so that more and more PEVs accumulate and herniations appear to represent a major log jam, as PEVs back up into the nucleoplasm. Our model to explain these observations suggests that the other putative viral or cellular de-envelopment factors can function at earlier stages of virus replication, but these factors are overwhelmed by the large numbers of HSV particles produced in the nucleus at late stages of infection. By this model HSV gB, gH/gL and US3 expedite the de-envelopment process, for which other viral and cellular factors are insufficient.

As far as we are aware, there have not been descriptions of cellular machinery that act to fuse NE membranes. Fusion of the inner and outer nuclear membranes appears not to be required for dissolution of the NE during mitosis. Rather, nuclear membranes are subsumed into the ER, which then vesiculates (reviewed in [94]). The formation of the de-envelopment fusion pore is topologically analogous to the formation of fenestrations in sheets of endoplasmic reticulum and to the creation of nuclear pores in the interphase nucleus, but the mechanism by which these fenestrations and pores are formed is currently unknown [94,95]. Intracellular transport vesicles fuse with acceptor molecules such as the plasma membrane, but the machinery involved, including Rab and SNARE proteins, are on the wrong face of the membrane to apply to virus particles or the ONM [96]. For fusion pore expansion and capsid release, it should be noted that the consensus sequence motif for pUS3 phosphorylation, RRRXS/T, overlaps that of cellular kinases including protein kinase A, Akt and protein kinase C [89,97,98,99]. Thus, it is possible that the US3-mediated phosphorylation can be assumed by cellular kinases, which are lacking in quantities or are less efficient. A similar picture may be proposed for gB and gH/gL, and other viral or cellular proteins may promote de-envelopment fusion.
